# Can incorporating genotyping data into efficacy estimators improve efficiency of early phase malaria vaccine trials?

**DOI:** 10.21203/rs.3.rs-3370731/v1

**Published:** 2023-09-22

**Authors:** Gail E. Potter, Viviane Callier, Biraj Shrestha, Sudhaunshu Joshi, Ankit Dwivedi, Joana C. Silva, Matthew B. Laurens, Dean A. Follmann, Gregory A. Deye

**Affiliations:** Biostatistics Research Branch, National Institute of Allergy and Infectious Diseases, National Institutes of Health; Clinical Monitoring Research Program Directorate, Frederick National Laboratory for Cancer Research; Malaria Research Program, University of Maryland; Malaria Research Program, University of Maryland; Institute for Genomic Sciences, University of Maryland School of Medicine; Institute for Genomic Sciences and Department of Microbiology & Immunology, University of Maryland School of Medicine; Center for Vaccine Development and Global Health, University of Maryland School of Medicine; Biostatistics Research Branch, National Institute of Allergy and Infectious Diseases, National Institutes of Health; Division of Microbiology and Infectious Diseases, National Institute of Allergy and Infectious Diseases, National Institutes of Health

**Keywords:** Vaccine efficacy, efficacy, genotyping, clone, molecular force of infection, molecular endpoints, early phase, trial efficiency, efficiency

## Abstract

**Background:**

Early phase malaria vaccine field trials typically measure malaria infection by PCR or thick blood smear microscopy performed on serially sampled blood. Vaccine efficacy (VE) is the proportion reduction in an endpoint due to vaccination and is often calculated as ${VE}_{HR}=1$ – hazard ratio or ${VE}_{RR}=1$ – risk ratio. Genotyping information can distinguish different clones and distinguish multiple infections over time, potentially increasing statistical power. This paper investigates two alternative VE endpoints incorporating genotyping information: ${VE}_{molFOI}$, the vaccine-induced proportion reduction in incidence of new clones acquired over time, and ${VE}_{C}$, the vaccine-induced proportion reduction in mean number of infecting clones per exposure.

**Methods:**

We used simulations and analytic derivations to compare power of these methods to ${VE}_{HR}$ and ${VE}_{RR}$ and applied them to three data sets: a Phase 3 trial of RTS,S malaria vaccine in 6912 African infants, a Phase 2 trial of PfSPZ Vaccine in 80 Burkina Faso adults, and a trial comparing *Plasmodium vivax* incidence in 466 Papua New Guinean children after receiving chloroquine + artemether lumefantrine with or without primaquine (as these VE methods can also quantify effects of other prevention measures). By destroying hibernating liver-stage *P. vivax*, primaquine reduces subsequent reactivations after treatment completion.

**Results:**

The RTS,S vaccine significantly reduced the number of clones at first infection, but PfSPZ vaccine and primaquine did not. Resampling smaller data sets from the large RTS,S trial to simulate phase 2 trials showed modest power gains from ${VE}_{C}$ compared to ${VE}_{HR}$ for data like RTS,S, but ${VE}_{C}$ is less powerful than ${VE}_{HR}$ for vaccines which do not reduce the number of clones at first infection. ${VE}_{molFOI}$ was most powerful in model-based simulations, but only the primaquine trial collected enough serial samples to precisely estimate ${VE}_{molFOI}$. The primaquine ${VE}_{molFOI}$ estimate decreased after most control arm liver-stage infections reactivated (which mathematically resembles a waning vaccine), preventing ${VE}_{molFOI}$ from improving power.

**Conclusions:**

The power gain from the genotyping methods depends on the context. Because input parameters for early phase power calculations are often uncertain, we recommend against these estimators as primary endpoints for small trials unless supported by targeted data analysis.

**Trial registrations:**

NCT00866619, NCT02663700, NCT02143934

## BACKGROUND

Phase 3 malaria vaccine trials generally use clinical malaria as an endpoint as this is a relevant measure of how a patient “feels, functions, or survives” (1). For small, early phase trials in malaria-experienced populations, rates of clinical malaria may be too low to have sufficient power. Outcomes with higher event rates, such as malaria infection detected by thick blood smear microscopy or PCR testing on serial blood samples are more feasible. Vaccine efficacy is often measured as ${VE}_{HR}=1$ – hazard ratio (2) or ${VE}_{RR}=1$ – risk ratio. The HR approach is generally more powerful than the risk ratio approach since it incorporates information on the timing of events and allows detection of a treatment effect even when all participants become infected during follow-up. It is more informative for a vaccine that is “leaky” (conferring partial protection on all individuals) than “all-or-nothing” (conferring complete protection on some individuals and no protection on others) (3, 4).

An alternative approach analyzes multiple infections per person during follow-up by comparing the incidence rates (IR) of infections between vaccine and control arms. Counting distinct infections is challenging when malaria infection is measured by thick blood smear microscopy performed on serially sampled bloods collected monthly and at sick visits because this information is insufficient to determine whether two consecutive positive results are distinct infections. Genotyping can be used to distinguish different infections over time and can also count synchronous infection with different strains as multiple infections. The number of new clones acquired per year has been referred to as the “molecular force of infection” (molFOI) and was correlated with clinical *P. falciparum* malaria (defined as febrile illness plus *Pf* parasitemia > 2500/μL) in Papua New Guinean children (5). Thus, an alternative efficacy measure is the proportion reduction in molFOI due to vaccination, measured as ${VE}_{molFOI}=1-\frac{\text{molFOIofvaccinees}}{\text{molFOIofcontrols}}$ This efficacy measure has been proposed as a new measure for vaccine efficacy trials (6, 7) but, to our knowledge, it has not been applied or tested in clinical vaccine trials, although it has been applied in observational studies: one study compared molFOI and infection clearing times between males and females to better understand higher malaria prevalence in males in cross-sectional samples (8), and another used molFOI in exploring possible mechanisms by which sickle cell trait (HbAS) protects against *Plasmodium falciparum* malaria (9).

${VE}_{molFOI}$ has the potential to increase power by incorporating information from multiple events, but it discards information from the timing of infections. A second efficacy measure incorporating genotyping information incorporates both time-to-event information and the number of clones present at the first infection (10, 11). This measure was developed in the context of HIV (with virions measured instead of clones) and has been applied to a Phase 3 trial of RTS,S malaria vaccine in 6912 infants. Although the authors denoted this measure ${VE}_{V}$ (V for “virion”), we will denote it ${VE}_{C}$ (C for “clone”). ${VE}_{C}$ is defined as the proportion reduction in number of infecting clones per exposure, due to vaccination. “Exposure” is the instantaneous exposure over time and is modelled as a completely flexible (unspecified) function of time. The only assumption is that the exposure function is identical in vaccine and control arms, which is reasonable for randomized controlled trials. To define ${VE}_{C}$, let $X^{*}$ denote the number of inoculated clones from a given exposure that develop into a blood stage infection, and let Z be a treatment indicator variable, so Z=1 for vaccinees and Z=0 for controls. Then, letting

$$\Delta =\frac{E(X^{*}|Z\;=\;1,\;exposed)}{E(X^{*}|Z\;=\;0,\;exposed)}$$,

the efficacy measure is:

$$VE_{C}=1-\Delta =1-\frac{E(X^{*}|Z\;=\;1,\;exposed)}{E(X^{*}|Z\;=\;0,\;exposed)}$$

We do not observe zero values of $X^{*}$ because we do not measure exposure events in malaria field trials and only detect clones that develop into blood stage infections. Let X denote the observed number of clones, so X is a truncated version of $X^{*}$ such that X>0. Through algebra, Follmann and Huang showed that we can estimate ${VE}_{C}$ as: ${\hat{VE}}_{C}=1-\frac{{\overset{‾}{x}}_{v}}{{\overset{‾}{x}}_{c}}HR$, where ${\overset{‾}{x}}_{v}$ and ${\overset{‾}{x}}_{c}$ are the mean number of clones at first infection in vaccinees and controls, respectively. While the estimand Δ is a ratio of untruncated population means, its estimator is a ratio of truncated means times the HR for the time to the first exposure that causes an infection. The intuition behind this is that if fewer exposures lead to infection in the vaccine group, then there are more unobserved zeroes in that group, which is mathematically reflected in the HR < 1 for the first infection. Note that for this measure, the numbers of clones are measured at the time of first infection. However, in contrast to a simple comparison of mean number of clones at first infection, ${VE}_{C}$ analyzes all participants (both infected and uninfected). ${VE}_{c}$ takes into account both the timing of the first infection and the number of clones for that infection.

**Figure 1:**
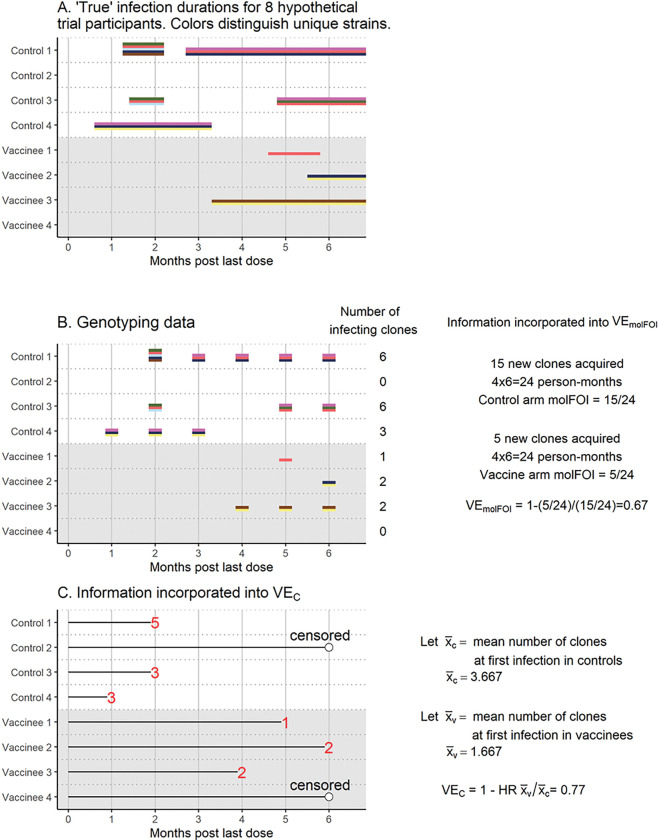
Infection durations for 8 hypothetical trial participants (Panel A), observed genotyping data and information contributing to ${VE}_{molFOI}$ (Panel B), and information incorporated into ${VE}_{C}$ (Panel C).

Figure 1 illustrates the information used and the calculation of the two VE measures incorporating genotyping data, ${VE}_{molFOI}$ and ${VE}_{C}$. Panel A shows “true” infection durations for 8 hypothetical vaccine trial participants, with colors distinguishing different clones. Panel B shows calculation of ${VE}_{molFOI}$. In this example, repeat observations of the same clone are counted as new infections if they were separated by at least one sample for which that clone was not observed. Panel C shows calculation of ${VE}_{C}$. Supplementary Figure A1 is an analogous figure for the same toy example showing the information incorporated into the two standard approaches, ${VE}_{RR}$ and ${VE}_{HR}$.

In this paper, we systematically explore the feasibility of these two VE measures in small trials by testing their operating characteristics in a comprehensive set of simulation studies and applying them to data from three randomized, placebo-controlled trials. The mean, variability, and statistical power of the VE measures are compared to standard VE estimators in model-based simulations and by resampling. We discuss implications of their adoption for the trial pipeline. Although we refer to these measures as VE (for “vaccine efficacy”), they can equally be considered and applied to trials of other malaria prevention measures.

## DATA

We analyzed the following three data sets:

1. RTS,S data: In this Phase 3 trial, participants in 7 African countries enrolled from March 2009 – Jan 2011 and were randomized 2:1 to receive either 3 doses of RTS,S/AS01 malaria vaccine or placebo (12) (NCT00866619). Clinical malaria from *Plasmodium* falciparum infection was tracked for 12 months, and thick blood smear microscopy was performed on samples collected at the first malaria episode. Serial blood samples were not collected. We analyzed 6912 participants aged 5–17 months who completed their dosing regimen and were included in the per-protocol population (11, 13). Of these, 2391 developed clinical malaria during the follow-up period, and genotyping results were obtained for 2089 (87%) of these first clinical malaria episodes. 908 (39%) of controls and 1181 (26%) of vaccinees experienced clinical malaria with non-missing genotyping results.
2. PfSPZ data: This Phase 2 trial included 80 Burkina Faso adults who enrolled in March 2017 and were randomized 1:1 to receive 3 doses of PfSPZ Vaccine or placebo (14) NCT02663700). Thick blood smear microscopy was performed on blood samples collected monthly and when ill with malaria symptoms to detect *P. falciparum* infection, and genotyping was performed on the first positive sample only. There were 37 first infections by thick blood smear microscopy: 14 of 39 (36%) vaccinees and 23 of 41 (56%) controls. Genotyping results were obtained for 33 of the 37 first infections (89%).
3. Primaquine data: This randomized controlled trial tracked molecular force of *P. vivax* blood stage infection in children randomized 1:1 to receive either blood + liver stage treatment (chloroquine (CQ) + artemether-lumefantrine (AL) + primaquine (PQ)) or blood stage treatment only (CQ + AL + placebo) at enrollment (15) (NCT02143934). *P. vivax* parasites can hibernate unobserved in the human liver (without causing symptoms in their host) for months or years and then “reactivate” to cause an observable and potentially symptomatic blood stage infection. The goal of primaquine treatment was to clear hibernating *P. vivax* parasites from the liver. Thus, *P. vivax* infections in controls included relapses and new infections, while in treated participants, new infections were assumed to be unchanged while a portion of relapses were prevented. The aim of the trial was to test whether the addition of primaquine to the CQ + AL regimen at baseline could reduce *P. vivax* infections in the subsequent 8-month follow-up period. 529 children aged 5–10 years enrolled from 17 August to 11 September 2009 in six villages in Maprik district, East Sepik Province, Papua New Guinea. Treatment was given over 28 days, with CQ for the first three days, PQ for 5 days per week, and AL for Days 11–13. After the end of the treatment period, blood was collected biweekly for 3 months, then monthly for 5 more months. We analyzed publicly available genotyping data from the 466 children who completed the full course of treatment (16, 17). Of these, 48% tested positive for *P. vivax* by PCR before study treatment, and 1% tested positive after treatment. The extent of missing genotyping results is unclear because the public data set provides limited information: it reports *P. vivax* parasitemia by PCR and the number of newP. vivax clones detected during an interval, so a positive *P. vivax* PCR can be consistent with zero new *P. vivax* clones (if the clones detected were previously observed). However, 31 (14%) of 229 first post-baseline infections were PCR-positive for *P. vivax* and had zero new P. vivax clones detected - an inconsistency between the two tests. Thus, we might expect that for about 14% of subsequent *P. vivax* PCR positive results, genotyping failed to detect the *P. vivax* clone that was present.

## METHODS

### Simulation Study 1

We simulated a malaria vaccine randomized controlled trial to compare power between the four VE methods. We assumed 1:1 randomization and a 168-day follow-up period. We simulated exposure events with equal rates in the two arms via a Poisson process, which is equivalent to sampling exponential times to exposure for each person and allows multiple events per person during follow-up. We define an “exposure event” as exposure sufficient to cause a blood-stage infection in a control participant, so the event may correspond to multiple infectious bites. Note that this is different from the standard conceptualization of exposure, which does not always lead to an infection in unvaccinated people. Each exposure event may transfer multiple *P. falciparum* clones, an assumption based on work suggesting that co-transmission of multiple clones from a single mosquito is more common than superinfection (infection with multiple clones from different mosquitoes) (18, 19). Each clone may then be blocked by the vaccine. We assumed that 10 clones are circulating in the community, and for each exposure event, the number of clones transferred (n_c_) was sampled from a Poisson random variable truncated to lie between 1 and 10. We performed separate simulations for a mean of 1, 2, or 3 clones transferred per exposure. Then, the identities of the n_c_ clones transferred during the bite were sampled from the set of 10 circulating clones with equal probability. Different simulations implemented different vaccine blocking mechanisms:

1. Scenario 1: Each clone was blocked independently with probability 0.5.
2. Scenario 2: A prespecified set including half of the circulating clones were blocked with probability 100% (when transferred to vaccinees), and the other half were never blocked.
3. Scenario 3: The prespecified set of half of the circulating clones were blocked with probability 75% (when transferred to vaccinees), and the other half were blocked with probability 25%.
4. Scenario 4: To estimate Type 1 error (the probably of incorrectly rejecting the null hypothesis when the null hypothesis is true), an ineffective vaccine was simulated.

For each clone that developed into a blood stage infection, an infection duration was sampled from an exponential distribution with a mean of 303 days for females and 167 days for males (8). We assumed biweekly sampling for 168 days and performed hypothesis tests for the four VE measures for each simulated trial. Power was estimated as the proportion of simulations in which the null hypothesis was rejected. Further details are in the supplemental material. The simulation was repeated with 500 circulating clones instead of 10 circulating clones, but we still assumed that a maximum of 10 clones could be transferred per exposure.

### Data Analysis

For each of the four VE methods, we calculated an efficacy estimate, 95% confidence interval (CI), and p-value for each data set. Risk ratios were estimated by modified Poisson regression (20) in the primaquine and RTS,S vaccine trials. This approach models the outcome as binary (infected vs. not infected) using a Poisson distribution, which gives a valid estimate of the risk ratio. Since a Poisson distribution is not a good fit for the Bernoulli distribution, variance estimates would be inaccurate by this approach, a problem corrected through the use of robust “sandwich” standard errors (20). In the PfSPZ Vaccine trial, the risk ratio was estimated based on Kaplan Meier estimated survival probabilities, as this method was prespecified as the primary analysis method. Their variances were calculated by Greenwood’s formula, and the delta method was applied to obtain the VE CI (21). In all trials, hazard ratios were from Cox regression with Wald tests, and ${VE}_{c}$ was calculated by estimating Δ as $\frac{{\overset{‾}{x}}_{v}}{{\overset{‾}{x}}_{c}}HR$, so ${\hat{VE}}_{c}=1-\frac{{\overset{‾}{x}}_{v}}{{\overset{‾}{x}}_{c}}HR$. Analogous to Cox regression, Δ is estimated on the log scale. The standard error for the estimator for log⁡(Δ) is given by the below formula (10):

$$SE_{\log\hat{\Delta }}=\sqrt{SE_{\hat{\log HR}}{\;}^{2}+\frac{s_{1}^{2}}{I_{1}{{\bar{X}}_{1}}^{2}}+\frac{s_{0}^{2}}{I_{0}{{\bar{X}}_{0}}^{2}},}$$

where ${SE}_{\hat{logHR}}$ is the standard error of the log hazard ratio from Cox regression, $s_{1}^{2}$ and $s_{0}^{2}$ denote the variances of number of clones at first infection in vaccinees and controls, respectively, ${\bar{x}}_{1}^{2}$ and ${\bar{x}}_{0}^{2}$ denote the mean number of clones at first infection and in vaccinees and controls, and $I_{1}$ and $I_{0}$ denote numbers of vaccinees and controls infected. Confidence intervals and p-values for ${VE}_{C}$ are calculated based on normality of the test statistic. In all trials, the ratio of molecular force of infection was estimated by quasipoisson regression with the total number of clones acquired over follow-up per person as the response variable and the log-transformed time-at-risk as an offset.

Time-at-risk was the end of follow-up in the primaquine data set because the genotyping results from serially sampled blood allow observation of repeat infections. Intervals of 42 days or more with no study visits were subtracted from time-at-risk for consistency with previous analyses (16). In the other two data sets, time-at-risk was truncated at the first infection since genotyping results from subsequent infections were not obtained. In calculating time-at-risk, we did not subtract time receiving antimalarial medication. Because time-at-risk in the RTS,S and PfSPZ Vaccine trials was based on the first malaria infection, there was likely little or no antimalarial medication received during the at-risk interval. In the primaquine trial, this analysis approach was taken for consistency with prior analyses. Although medication data during the follow-up period is not available in the public data set, clinical episodes (defined as fever and a positive malaria infection by light microscopy) are in the data set, and there were few in this trial: 27 controls (12%) and 18 PQ recipients (8%) experienced clinical malaria. In the primaquine data set, 31 (14%) first infections were PCR positive for *P. vivax* but had no *P. vivax* clones reported. These were analyzed as zero clones when calculating ${VE}_{molFOI}$. Of these 31, 27 had zero and 4 had missing new clones; these were analyzed as zero and missing, respectively, when calculating the mean number of clones at first infection. A sensitivity analysis was performed imputing each of these events to have 1 new *P. vivax* clone. In the Burkina Faso data set, 4 first infections were missing a genotyping result. These were analyzed as no new clones when calculating ${VE}_{molFOI}$ and as missing when calculating the mean number of clones at first infection. In the RTS,S trial, only clinical malaria events with non-missing genotyping information were analyzed, for consistency with previous analyses (11).

### Simulation Study 2

The purpose of this simulation is to assess whether the three data sets are consistent with the model underlying the simulation process. The model operationalizes the vaccine mechanism as a per-clone blocking probability. From this, reductions in all outcomes (risk of infection, hazard ratio, mean number of clones per infection, and molFOI) are affected simultaneously, so treatment effects on all outcomes are correlated. We recalibrated the inputs to the initial simulation study to match the probability of at least one infection during follow-up in each group and the mean number of clones per infection in the control group for each of the three trials analyzed. The inputs adjusted were the exposure rate, the mean number of clones transferred per exposure, and the clone blocking probability. The mean number of clones per infection in the vaccine group is induced by the distribution of clones in the control group, the clone blocking probability, and the blocking mechanism. The exposure rate was mathematically derived (assuming exponential time-to-event distributions) so that the simulated control group infection rate matched the observed rate. Blocking probabilities were then estimated by looping through different blocking probability values until the simulated treatment group event rate matched the observed rate. Table 1 shows these infection rates for each trial and the values of input parameters used to simulate them. The actual total number of clones circulating in the community was not available, so we assumed 50 circulating clones in each community with a maximum of 10 clones transferred per exposure. For each set of inputs, we simulated 1000 trials of size 500 and tracked the average VE estimator values, infection rates in each arm, and the mean and variance of the number of clones at first infection in each arm.

### Simulation Study 3 and Analytic Power

Because the Phase 3 RTS,S data set is very large, it provides a population from which we can resample smaller trials, which are hypothetical early phase trials. We resampled 10,000 trials of size 80, 150, and 250 from the RTS,S data set with equal numbers of participants in each arm. ${VE}_{HR}$ and ${VE}_{C}$ were calculated with 95% confidence intervals, and power was estimated as the proportion of simulations for which the interval excluded zero. Since the RTS,S trial had a lower event rate than what we usually see in early phase trials, we then imposed a higher event rate by first simulating infection status for each control by tossing a weighted coin with infection probability 0.6, and doing the same for vaccinees with infection probability 0.3. For each infected vaccinee, the paired values of the time-to-event and number of clones at first event were sampled from the set of infected RTS,S vaccinees. For each uninfected vaccinee, the time to censoring was sampled from the set of uninfected RTS,S vaccinees. Values were sampled analogously for controls. To provide further context for Simulation Study 3, we derived analytic power formulas for two of the efficacy methods: ${VE}_{HR}$ and ${VE}_{C}$. These experiments and derivations were not performed for ${VE}_{molFOI}$ because the information in the RTS,S data set for this estimator is limited due to the lack of serial sampling.

## RESULTS

**Figure 2:**
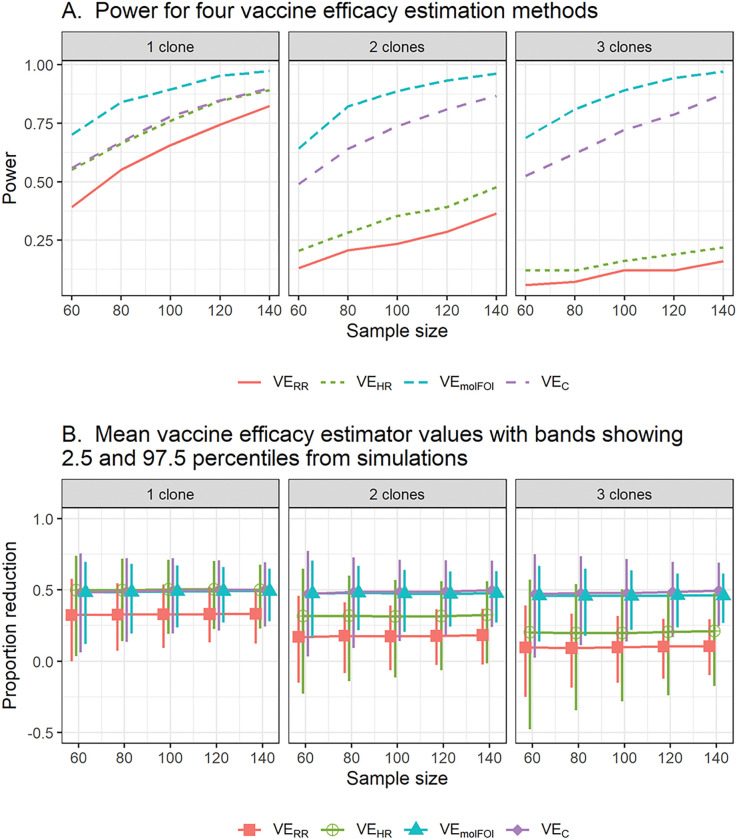
Estimated power by the four different VE methods by mean number of clones transferred per exposure (Panel A) and mean VE estimator values across simulations (Panel B) with variability represented by 2.5^th^ and 97.5^th^ percentiles. VE methods were calculated for the same sample sizes; the displayed intervals are staggered to distinguish them visually.

Simulation Study 1 found that Type 1 error is controlled for all methods and shows similar power for each method under the three vaccine-blocking scenarios (Supplementary Figure A2). This is because while the different scenarios implement different preferential blocking for different clones, the mean number of clones is reduced by the same amount. An exception is that for small sample sizes and large average number of clones transferred per exposure, the molecular force of infection method is more powerful in Scenario 2 (perfect blocking of half the clones and no blocking of the other half) than in other scenarios. That is because we assumed 10 circulating clones and reducing this to 5 among vaccinees reduces the variability of the number of clones, which reduces the variability of the VE estimator. Because the three scenarios of interest gave similar power, we display only power for Scenario 1 in Fig. 2 to facilitate comparison between methods. The simulations result in an infection rate of 75% in the control arm and 50% in vaccinees when a single clone is transferred per exposure. All panels show that the hazard ratio approach is more powerful than the risk ratio approach. When a single clone is transferred per exposure, ${VE}_{HR}$ and ${VE}_{C}$ perform identically since the mean number of clones at first infection is 1 in both groups. When 2 clones are transferred per exposure (on average), the value of the HR is reduced because the vaccine must block two clones (each with probability 0.5) to prevent an infection, so ${VE}_{HR}$ loses power, as does ${VE}_{RR}$, but the genotyping methods do not lose power. This pattern continues when the mean number of clones per exposure is increased to 3. The lower panel shows that the expected values of estimators are constant across sample sizes, but variability of the estimator decreases as the sample size increases.

When the simulation was repeated with 500 circulating clones, power and VE estimates were nearly identical to those obtained when we had 10 circulating clones (Supplementary Figures A3 and A4) for each scenario, estimator, and mean number of clones. This is because the mean number of clones is reduced by the same amount, regardless of which clones were transferred. We found only one difference from the previous set of simulations: with 500 circulating clones, ${VE}_{molFOI}$ did not have higher power in Scenario 2 than in the other scenarios because there is no truncation on the number of clones vaccinees can experience. In general, communities with more circulating clones tend to have higher transmission. We obtained similar results in these two experiments because, in our simulation model, the parameters driving differences in transmission levels are the exposure rate and the mean number of clones transferred per exposure, and these had the same values for the two simulations. For this reason, the simulations calibrated to each of the three analyzed trials (which all assumed 50 circulating clones) capture differences in community transmission levels (by varying these two parameters) even if these communities actually have different numbers of circulating clones.

**Figure 3:**
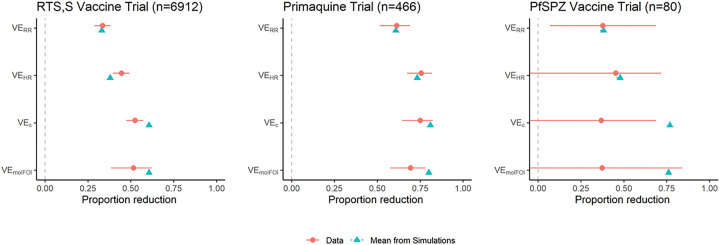
VE estimates (red circles) with 95% confidence intervals from three clinical trials. The blue triangles show expected values of the VE estimates from simulation models calibrated to match the event rates in both arms and the mean number of clones at first infection in controls.

Figure 3 displays VE estimates and 95% CIs for the three trials analyzed. Numeric values are in Supplementary Table A2, and Supplementary Table A3 provides summary statistics for each trial. The blue triangles show the average VE values induced by the simulation model that matches the event rates in each group and mean number of clones at first infection among controls. 95% confidence intervals for the mean VE from simulations are smaller than the plotting symbols. Note that these are different from the percentiles shown in Fig. 1, which displays variability between trials of the observed VE. The intervals in Fig. 3 display uncertainty in our estimation of the true VE induced by the model (which can be made arbitrarily small by increasing the number of simulations). By design, the mean ${VE}_{RR}$ from simulations is very close to the observed ${VE}_{RR}$. The leftmost panel summarizes RTS,S trial results, which show that ${VE}_{HR}$ is larger than ${VE}_{RR}$, with a confidence interval farther from the null value of zero, and that ${VE}_{C}$ and ${VE}_{molFOI}$ are similar and are both higher than ${VE}_{HR}.\;{VE}_{C}$ has a confidence interval farther from the null value than ${VE}_{HR}$, but ${VE}_{molFOI}$ does not and has a wider interval. Information in this trial for ${VE}_{molFOI}$ is limited because only the first infection was genotyped. The CIs are wider in the primaquine trial and much wider in the PfSPZ Vaccine trial due to the smaller sample sizes of these trials. In all three trials, the blue triangles show that the values of ${VE}_{C}$ and ${VE}_{molFOI}$ predicted by the simulation model are higher than those estimated from the data. Moreover, the model-predicted value of ${VE}_{C}$ is significantly higher in RTS,S and PfSPZ (as the triangle lies to the right of the CI), but not the primaquine trial. The model-predicted value of ${VE}_{molFOI}$ is significantly higher in the primaquine trial. The discrepancy between the simulation-predicted VE values and those from the data suggest that our model tends to overestimate the VE measures incorporating genotyping data, which would cause it to overestimate the power gain from these approaches.

We performed the following analyses to explore possible reasons for the discrepancies between the simulation model results and estimates from the data:

### RTS,S Trial

We compared summary statistics from the RTS,S data set to those from simulations. Although the simulations match the event rates in each group, they predict a larger reduction in mean number of clones at first infection (from 2.26 in vaccinees to 1.44 in controls; while the actual controls had a mean of 1.94 clones (95% CI [1.86, 2.02]) (Supplementary Table A4). The simulations also underestimate variability of the number of clones. We revised the simulation to match the entire distribution of clones in controls instead of using a truncated Poisson distribution that matches only the mean. This revision only slightly reduced the discrepancy between the data and the simulation summaries, so is not the source of the difference (Supplementary Table A4). Although the ordering of the RTS,S VE estimates is consistent with the simulation outputs (unlike the other estimates for the trials), the discrepancy between the data and the simulation summaries indicate that the simulations overestimate power for this data structure.

### Primaquine Trial

The treatment tested in this trial prevents reactivations of liver stage infections and is not expected to prevent new infections or reinfections from an outside source. Thus, the mechanism is different from the simulation model we assumed for malaria vaccines. However, it is similar to the second scenario we considered - a vaccine that blocks half of all clones with 100% probability and no blocking for others. Although the set of clones to be blocked would differ between individuals in this trial, the model might be an adequate approximation.

The observed treatment effect in this trial on molFOI will eventually decline because after all liver-stage infections in controls have been reactivated, susceptibility to new *P. vivax* infections will be similar between controls and treated participants. The declining treatment effect is mathematically similar to a waning VE for a malaria vaccine, although the biological mechanism is different. Supplementary Figure A5 shows monthly molFOI by treatment arm during follow-up. Control arm *P. vivax* molFOI surged in Months 2 and 3 post-enrollment, but the treated arm did not surge similarly. Hofmann et al. (16) conjectured that the surge could be due to a triggering of relapses from the blood-stage treatment or that relapses may have occurred soon after treatment (while antimalarial drug levels were low), then were suppressed from detection as drug levels rose and were finally detected after drug levels waned. The molFOI for this group dropped steeply in Month 4 but remained larger than the molFOI in the treated arm for the rest of follow-up (Supplementary Figure A5), possibly because reactivations continued at a slower rate among controls. This pattern is consistent with models of *P. vivax* recurrence in other studies (22–24). Further modeling to understand the mechanisms influencing *P. vivax* reactivation is needed and is beyond the scope of this paper. Our simulation model does not implement waning, and the power gain from the molecular force of infection approach is less when waning occurs because this measure incorporates information from all infections and does not incorporate the time to first infection.

${VE}_{C}$, however, is based on the time to first infection and number of clones present at first infection. The mean number of clones at first infection was 1.46 in controls and 1.49 in treated participants, making ${VE}_{C}$ smaller than ${VE}_{HR}$. When we repeated the analysis with only the first three months of follow-up, the genotyping VE estimates were larger than ${VE}_{HR}$ with confidence intervals farther from the null value (Supplementary Table A2), but the mean numbers of clones at first infection were 1.51 and 1.44 in control and treated arms, which do not differ significantly or substantively. Our simulation model induces a relationship between the risk reduction and the mean ratio, which is why the simulation-predicted value for ${VE}_{C}$ is greater than that for ${VE}_{HR}$. The combination of a dramatic risk reduction and null mean ratio observed in this trial are not what we expect from the model, but the model-predicted values of both ${VE}_{C}$ and ${VE}_{HR}$ are within the confidence intervals generated from this trial.

Our simulations used exponential infection durations, which may result in too many short durations, giving too many opportunities for new infections. To test if this was causing us to overestimate power, we revised the original simulation study assuming all infections lasted longer than the follow-up period. Results were similar (Supplementary Tables A5 and A6), so this assumption was not the root of the problem. Finally, a sensitivity analysis in which new post-baseline *P. vivax* infections with zero new *P. vivax* clones detected were imputed to have 1 new clone gave nearly identical results to our primary analysis (Supplementary Table A2).

### PfSPZ Vaccine Trial:

The small sample size of this trial creates a large amount of uncertainty in estimates. The mean number of clones at first infection was higher among vaccinees than controls (3.46 vs. 3.00), but the difference was not statistically significant (t-test p = 0.30). The ratio of mean number of clones at first infection for vaccinees to controls is 1.15. In the RTS,S trial, we saw the opposite pattern: there were significantly fewer mean number of clones in vaccinees (1.94) *vs*. controls (2.26) at first infection. When we resampled 10,000 data sets of size 80 from the RTS,S data set, we found that vaccinees have fewer clones than controls in only 71% of resampled data sets. They have more clones than controls in 29% of resamples, and the ratio of means is ≥ 1.15 in 14% of resampled data sets. This means that 14% of trials of size 80 of a vaccine that reduces the number of clones at first infection as much as the RTS,S vaccine did will give a ratio of means ≥ 1.15. Therefore, it is difficult to draw a conclusion about what the PfSPZ Vaccine is doing, in terms of number of clones at first infection, from this trial.

### Simulation Study 3

Our third simulation study entailed resampling data sets of smaller sizes from the RTS,S data to estimate power with a realistic data structure and no model assumptions. The first three rows of Table 2 show that power estimated by this approach is modestly higher with ${VE}_{C}$ than ${VE}_{HR}$. The fourth row shows power estimated by imposing artificially higher event rates but preserving the time-to-event distributions among people who became infected as well as the distribution of number of clones. With these higher event rates and a higher risk ratio, power is lost rather than gained when genotyping data is added. This is because although the addition of genotyping data can add information, it also adds variability to the estimator because the two means are being estimated, so the confidence intervals are wider.

The increased uncertainty by adding genotyping information can be seen directly from the formula for the standard error of the estimator derived in (10). The estimator is ${\hat{VE}}_{c}=1-\frac{{\overset{‾}{x}}_{v}}{{\overset{‾}{x}}_{c}}HR=1-\hat{\Delta }$. As noted previously, the standard error for the estimator for log⁡(Δ) is:

$$SE_{\log\hat{\Delta }}=\sqrt{SE_{\hat{\log HR}}{\;}^{2}+\frac{s_{1}^{2}}{I_{1}{{\bar{X}}_{1}}^{2}}+\frac{s_{0}^{2}}{I_{0}{{\bar{X}}_{0}}^{2}}}$$

**Figure 4:**
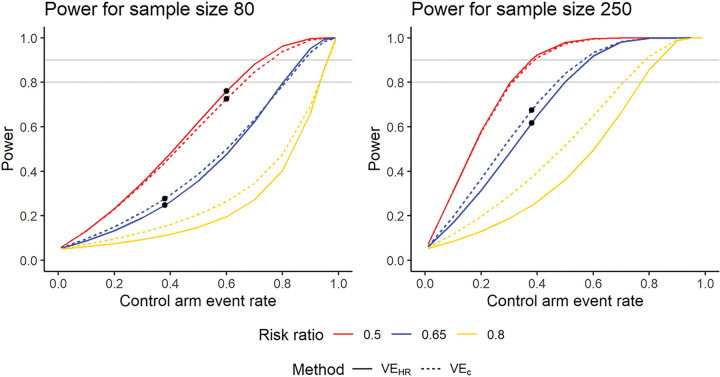
Power curves for sample sizes 80 and 250 for ${VE}_{HR}$ and ${VE}_{C}$. The calculations assume exponential time-to-event distributions, and the means and standard deviations of number of clones at first infection ineach group match those in the RTS,S data. The black dots correspond to combinations of input parameters in Table 2. The analytic power estimates differ slightly from those in Table 2 because the RTS,S time-to-event distribution is not exponential, but they show the same general pattern.

Thus, the variance of the estimator for log⁡(Δ) is always greater than the variance of the log-transformed hazard ratio. Since 95% confidence intervals for the log-transformed estimands are calculated as Estimate ± 1.96 $\sqrt{Var\;(\text{Estimator})}$, the above variance formulas mean that the confidence interval for log⁡(Δ) is always wider than that for log(HR). Adding genotyping information into the efficacy estimator can improve power when the reduction in mean clones is large and added variability is small, but can decrease power when the reduction in mean clones is small and added variability is large. We derived analytic formulas for power in the Supplementary Appendix to try to establish a threshold beyond which power is lost rather than gained by adding genotyping information. However, these formulas show that power depends on the sample size in each group, the numbers infected in each group, the hazard ratio, and the means and standard deviations of numbers of clones at first infection in each group. The threshold at which ${VE}_{C}$ starts to have less rather than more power than ${VE}_{HR}$ depends on all these parameters and can’t be reduced to a simple formula. Figure 4 shows power curves for sample sizes 80 and 250 for three different risk ratios. To reduce the number of input parameters for the power curves and create a simpler visualization, we assumed an exponential distribution for the time to first infection. In this case, the HR is defined by the infected proportions in each group as $HR=\frac{log\left(1-p_{1}\right)}{log\left(1-p_{0}\right)}$. The simulation results in Table 2 and power curves in Fig. 4 show that with a greater risk reduction, the extra information from the relatively small mean reduction observed in RTS,S is outweighed by the additional uncertainty added from estimating two means.

The derivations in the Supplementary Appendix show that the threshold reversing the power gain from ${VE}_{C}$ does not depend on the sample size. The threshold occurs in the same place in the left and right graphs. For a risk ratio of 0.65, the threshold occurs at a control arm event rate of 0.74. The magnitude of the power difference between the two methods does depend on the sample size, however.

## DISCUSSION

We have compared two new malaria vaccine estimators incorporating genotyping data to the standard estimators by evaluating their operating characteristics in simulations and applying them to data from three trials. Both genotyping estimators were more powerful than standard estimators in model-based simulations, but analysis of the three trials suggests that the simulation model overestimates power. Despite this limitation, the simulation model gave insight into the performance of the two new VE methods. They control Type 1 error in all settings considered. In addition, the simulations showed that power from the genotyping methods generally did not differ when the vaccine blocked certain clones differentially as long as it blocked the same number of clones on average. An exception is a vaccine that completely blocks a subset of clones when there are a relatively small number of circulating clones, because this can reduce the variability of the estimator. The simulations also showed that the potential power gain increases with number of clones transferred per exposure, so there is less potential benefit in communities with few circulating clones, and ${VE}_{C}$ showed no benefit with a single clone transferred per exposure. Communities with more circulating clones tend to have a higher exposure rate and more clones transferred per exposure. When these parameters are fixed, however, results are similar if the number of circulating clones increases.

Our second simulation study entailed resampling smaller trials from the RTS,S vaccine trial. This does not rely on any model assumptions. This study showed that ${VE}_{C}$ has moderately higher power than ${VE}_{HR}$ in some realistic scenarios and lower power in others because there is a balancing act between adding data that can be informative about the vaccine and the additional variability in estimation contributed by estimating the two means. In data resembling the RTS,S trial, we found a power gain of 3 to 4 percentage points in small trials. The reduction in mean number of clones outweighs the increase in variability of the test statistic, and power is gained. When the risk reduction is larger than that seen in RTS,S, incorporating the mean reduction adds relatively more variability, and power is lost. In early phase trials, we typically have a lot of uncertainty around the inputs going into the power calculation, so we recommend against this estimator as a primary efficacy approach.

There is less information in the three trials for understanding ${VE}_{molFOI}$ than ${VE}_{C}$ since only one of the trials included genotyping results from serially sampled blood, and this one was a treatment trial rather than a vaccine trial and so it has a different mechanism of action. ${VE}_{molFOI}$ performed well in power simulations, but the treatment trial had a declining treatment effect for this endpoint, and the temporal decline decreases the size of the estimator and makes this method less powerful than ${VE}_{HR}$. In the RTS,S trial, the value of ${VE}_{molFOI}$ was higher than that of ${VE}_{HR}$, but the confidence interval was much wider since only the first infection was genotyped. In RTS,S, the mean number of clones at first infection was significantly lower in vaccines than controls (1.94 vs. 2.26; p < 0.0001, t-test), unlike the other two trials. Furthermore, trials of RTS,S administered with AS02A or AS01B adjuvant also found the vaccine to reduce number of clones at first infection in children under five in Mozambique (25) and to reduce number of clones in adults in western Kenya (26). Depending on the extent of waning of the RTS,S vaccine in 12 months and the ratio of mean number of clones at each infection, it is possible that the ${VE}_{molFOI}$ would be more powerful than ${VE}_{HR}$ in trials with similar control arm event rate and vaccine mechanism but with genotyping results from serial samples. In the PfSPZ Vaccine trial analyzed in this paper, vaccinees had more clones at first infection than controls (3.46 vs 3.00, p = 0.30), but the difference was not statistically significant, and simulations showed that this difference is a plausible value even if the true effect is actually the reduction observed in the much larger RTS,S trial. The sample size of the PfSPZ trial is too small to draw conclusions about ${VE}_{molFOI}$. Genotyping measures from serially sampled blood from a much larger malaria vaccine trial would help determine if ${VE}_{molFOI}$ may be helpful.

A major consideration of early phase endpoints is their performance in distinguishing which vaccines will be most likely to demonstrate an effect on the clinical endpoint of interest when they are tested in later phase trials. If a reduction in the number of clones does not also reduce the risk of clinical malaria, then adding genotyping information to our VE estimators is just adding noise that could obscure our ability to distinguish performance between different vaccine candidates in early phase trials rather than adding information that will help differentiate their performance. Many individuals with polyclonal infections do not have symptomatic malaria, so vaccines which reduce their number of clones may have larger VE estimators by the genotyping approaches than other vaccines, even though they may not do as well at preventing clinical malaria. Therefore, even if ${VE}_{molFOI}$ can increase the size of VE estimators for some vaccines, the risk of adopting this measure as a primary outcome should be carefully considered. It would be safer to include it as a secondary or exploratory outcome to better understand its relationship to clinical malaria as well as to the epidemiology and natural history of the disease. Such data could also help with understanding the mechanism of action of vaccines and treatments.

A necessary condition for either of these genotyping endpoints to be a good surrogate is a positive relationship to clinical malaria. Molecular force of infection was positively related to clinical malaria in studies of infants and children (5, 16) and of a broader age population (27). For ${VE}_{C}$, a positive relationship would be needed between clinical malaria and the number of clones (also called the multiplicity of infection, MOI) at first infection, and evidence for this is mixed. The RTS,S trials cited earlier (including the one that we analyzed) support a positive relationship, as do some studies of children (28, 29). However, one study found similar MOI between asymptomatic and symptomatic participants (30), and others found lower MOI in symptomatic than asymptomatic infections (31, 32). This could be because the higher density of one dominating strain driving the infection makes it harder to detect the presence of other strains. Alternately, people with higher immunity, and hence asymptomatic, may have higher MOI at first symptomatic infection since their immunity allowed them to control infection with clones similar to others acquired earlier in life. Further study is needed.

An association with clinical malaria is a necessary but not sufficient condition for a good surrogate outcome. A surrogate must also lie in the disease process pathway on which the intervention acts and capture all relevant on-target and off-target effects of the intervention on the disease process (1). Generally, this comprehensive understanding of the disease process is impossible, and surrogacy is instead validated by a meta-analysis relating the treatment effect on the surrogate to the treatment effect on the clinical outcome in multiple trials (33). We acknowledge that this validation is not typically required for early phase trial endpoints; rather we raise these issues to point out the risk of substituting one early-phase trial endpoint with another.

Although infection detected by microscopy is a standard early phase outcome, its relationship to clinical malaria as a surrogate is also unclear since the development of disease from blood-stage infection depends on immunity and exposure, which differ between individuals but tend to be correlated since past exposure is a predictor for current immunity. For example, a vaccine that blocks blood stage infections only in the most robust individuals (who would not have developed clinical malaria anyway), could reduce malaria infection but not reduce clinical malaria in individuals. Genotyping data can help explain these relationships. For example, one study found that antibody levels were associated with increased risk of clinical malaria in children aged 1–4 in Papua New Guinea, but adjustment for molFOI removed most of the association, indicating that the antibody levels were a proxy for exposure (34). When the analysis was repeated on a cohort aged 5–14 years, antibody levels were associated with protection from clinical malaria, suggesting that a threshold level of antibody levels is needed to reduce the risk of disease. Collection and analysis of genotyping data on a large scale is needed to further disentangle these relationships (35).

This study has limitations. Although our simulation model gave insight into the performance of the two new estimators, it overestimated the increase in the VE value for the VE methods incorporating genotyping data. Thus, it may also overestimate the power gain from these methods. The model-based simulations calibrated to the RTS,S trial estimated a larger reduction in number of clones at first infection than what was observed. The simulation model assumed a purely leaky vaccine, meaning that the vaccine reduces the probability of infection equally for all vaccinated participants. The RTS,S vaccine may instead be a combination of all-or-nothing and leaky. Models have been developed to test whether a vaccine is all-or-nothing, leaky, or combination, but require genotyping data from multiple malaria exposures (36), which were not available in this data set. Another possible explanation for the discrepancy is that our model assumed equal distributions of the clones in circulation, perfect sensitivity in detecting all clones, and no competition between clones. It is possible that in infections with larger numbers of clones (e.g., in controls), the clones present in lower frequencies may be harder to detect. “Competitive release”, in which clearance of some clones creates space for others to flourish (37), could also water down the vaccine-induced reduction in number of clones. The observed 10–15% missing genotyping results in our data sets indicates imperfect sensitivity, which could explain some of the discrepancy. The simulation model did not include antimalarial treatment blackout periods (time not-at-risk during antimalarial treatment). For an effective vaccine, more controls will become ill than vaccines, so subtracting blackout periods would reduce the time-at-risk (the denominator for molFOI) more for controls than for vaccinees, inflating the treatment effect estimates. However, this approach is consistent with our analysis of the trials, and in the RTS,S and PfSPZ Vaccine trials, antimalarial medication was likely not given before the first detected malaria infection.

Another limitation is that two of the three trials measured genotyping data only at the first observed infection by thick blood smear microscopy. Only the primaquine trial followed the optimal sampling scheme for the proposed VE estimators: PCR testing of serially sampled blood with genotyping performed on all PCR positive samples. Because microscopy is less sensitive than PCR, some first infections may have been missed. Since genotyping was not performed on repeated samples, power for ${VE}_{molFOI}$ was limited in these two trials, as was information about the molFOI time trend.

A third limitation is that the primaquine trial was not a vaccine trial, but these genotyping efficacy estimators could be useful for trials of any malaria prevention intervention. The primaquine treatment mechanism differed from our simulation model, but it resembles the simulation scenario in which some clones were always blocked. The deviations between model-predicted and data-estimated statistical summaries were similar between the three trials, suggesting that the operating characteristics of the estimators are similar for this type of treatment trial and for vaccine trials. Since the primaquine treatment was completed before the follow-up period began, waning was inevitable after all sequestered *P. vivax* infections among controls were reactivated. The similar (rather than improved) performance of ${VE}_{molFOI}$ and ${VE}_{C}$ in this trial compared to the standard estimators was due in part to waning. This performance can be expected for any intervention with waning, including vaccines in development.

Despite these limitations, this is the first paper to explore the potential benefit of incorporating genotyping data into VE measures for early phase trials by systematically studying their operating characteristics in a range of settings and vaccine mechanisms. Our analysis suggests that ${VE}_{C}$ is not likely to systematically improve upon the widely used ${VE}_{HR}$ in early phase malaria vaccine trials. Because ${VE}_{molFOI}$ showed the most promise in simulations and our data to evaluate this measure was limited, this measure merits further exploration, which would require genotyping data from serially sampled blood for one or more large malaria vaccine trials. Such data would be used to explore multiple scientific questions (38) and to determine the scenarios in which ${VE}_{molFOI}$ will be most useful and informative.

## Figures and Tables

**Table 1:** Target infection rates and inputs used to generate them in Simulation Study 2

	Parameter	RTS,S/AS01 trial	Primaquine trial	PfSPZ Vaccine trial
Infection rates observed in each trial	Proportion of controls infected during follow-up	0.39	0.71	0.56
	Proportion of subjects in treatment arm infected during follow-up	0.26	0.28	0.36
Inputs to simulation study	Exposure rate	1/741	1/182	1/204
	Clone blocking probability	0.61	0.80	0.76
	Mean number of clones at first infection in control group	2.26	1.46	3.00
	Follow-up duration	12 months (364 days)	8 months (224 days)	6 months (168 days)

**Table 2:** Power estimated by resampling smaller trials from the RTS,S data. The first three rows show estimated power from direct resamples of the RTS,S data. The fourth row shows power from an artificially imposed higher control group event rate and larger risk ratio reduction but matches the mean clones and time-to-event distributions among infected participants in each group. Because time-to-event distributions match only those among infected RTS,S participants but fewer people escape infection than in the RTS,S data, the hazard ratio for the fourth row does not match that in the RTS,S data.

Control arm event rate	Vaccine arm event rate	Sample size	VE_HR_ power	VE_C_ power
0.38	0.26	80	0.29	0.33
0.38	0.26	150	0.52	0.56
0.38	0.26	250	0.75	0.78
0.60	0.30	80	0.85	0.80

## Data Availability

RTS,S Data: The authors do not have permission to share this data set, but the results in this manuscript are consistent with previously published analyses (11). The supplementary material for that publication includes code and a fake data set (with the same structure as the real data set) so that readers can test the statistical methods on the fake data set (11). PfSPZ Data: Upon acceptance, an analysis data set will be shared in a public repository, and the link will be included here. Primaquine Data: The data are publicly available (17).
